# Relationship of the Iliac Crest Height with Subsidence After Oblique Lateral Interbody Fusion at L4–5: A Quantitative and Categorical Analysis

**DOI:** 10.3390/jcm13206223

**Published:** 2024-10-18

**Authors:** Jae-Hyuk Yang, Kun-Joon Lee, Seung-Yup Lee, Hyung-Rae Lee

**Affiliations:** 1 Department of Orthopedic Surgery, Korea University Anam Hospital, Seoul 02855, Republic of Korea; kuspine@korea.ac.kr (J.-H.Y.); yup1019@naver.com (S.-Y.L.); 2 College of Medicine, Korea University, Seoul 30019, Republic of Korea; slcjohn@daum.net

**Keywords:** oblique lateral interbody fusion, OLIF, iliac crest height, subsidence, lumbar spine, spinal surgery, cage obliquity

## Abstract

**Background:** This study aimed to evaluate the impact of iliac crest height on clinical and radiological outcomes following oblique lateral interbody fusion (OLIF) at the L4–5 level. **Methods:** Data of patients who underwent single-level OLIF at the L4–5 level for degenerative spinal stenosis were retrospectively analyzed. The patients were categorized into three groups based on their iliac crest height measured relative to the L4 and L5 pedicles. Categorical and quantitative analyses, including univariate and multivariate logistic regressions, were performed to identify subsidence predictors. Clinical outcomes, including visual analog scale scores for back and leg pain, were assessed over a minimum 2-year follow-up. **Results:** No significant differences in cage obliquity were observed across the iliac crest height groups (axial angles, *p* = 0.39; coronal angles, *p* = 0.79). However, subsidence was significantly more common in patients with higher iliac crest heights, particularly at crest level III, where the subsidence rate reached 43% (*p* = 0.01). Subsidence was predominantly associated with damage to the L5 endplate, which occurred in 83% of subsidence cases at crest level III. A cutoff value of 12 mm for iliac crest height, above which the risk of subsidence significantly increased, was identified (AUC = 0.688, *p* = 0.042). **Conclusions:** Iliac crest height is a critical factor for predicting subsidence following OLIF at the L4–5 level. Surgeons should consider alternative strategies and meticulous preoperative planning in patients with an iliac crest height ≥ 12 mm to reduce the risk of adverse outcomes. Further studies are needed to validate these findings and to explore their long-term implications.

## 1. Introduction

Oblique lateral interbody fusion (OLIF) is a minimally invasive spinal surgical technique that was first introduced by Mayer in 1997 [1,2]. OLIF offers several advantages over traditional posterior lumbar interbody fusion surgery [3]. In particular, OLIF reduces the risk of posterior muscle damage because it avoids damaging the posterior musculature and ligaments [4]. Additionally, OLIF allows for the anterior insertion of large lordotic cages, which can enhance spinal alignment and stability [5,6,7]. The minimally invasive nature of OLIF also contributes to reduced blood loss, a shorter operative time, and faster postoperative recovery, making it an increasingly popular option for lumbar fusion, particularly at the L4–5 level [5,7]. OLIF is performed through a retroperitoneal approach that involves accessing the disc space between the peritoneum and psoas muscle, which provides a direct path while minimizing muscle disruption. This approach reduces the complications associated with muscle retraction and offers a favorable trajectory for cage insertion [5].

However, performing OLIF at the L4–5 level can be challenging, especially in cases where the L4–5 disc space is deep below the high iliac crest [1]. An elevated iliac crest height can obstruct the surgeon’s view through the incision site and limit access to the L4–5 disc space, making it difficult to achieve the proper angle for the procedure. This difficulty is often exacerbated in patients with lumbosacral transitional vertebrae, where anatomical variations often accompanied by a high iliac crest, and deep-seated L4–5 levels can further complicate the surgical approach [8]. Previous studies have emphasized the need for caution when performing OLIF L4–5 procedures when the iliac crest is high; however, these discussions are largely theoretical [2,8,9,10]. Unfortunately, no studies have quantitatively analyzed how varying iliac crest heights specifically impact OLIF outcomes. We hypothesized that issues such as cage obliquity and endplate damage leading to subsidence could be significant concerns owing to their potential adverse effects on the overall success rate of spinal fusion and patient recovery.

Therefore, this study aimed to analyze the clinical and radiological outcomes of OLIF at the L4–5 level in relation to the height of the iliac crest. Specifically, this study compared the incidence of adverse outcomes such as cage obliquity and subsidence across different iliac crest height grades.

## 2. Materials and Methods

### 2.1. Study Design and Patients

**Figure 1:**
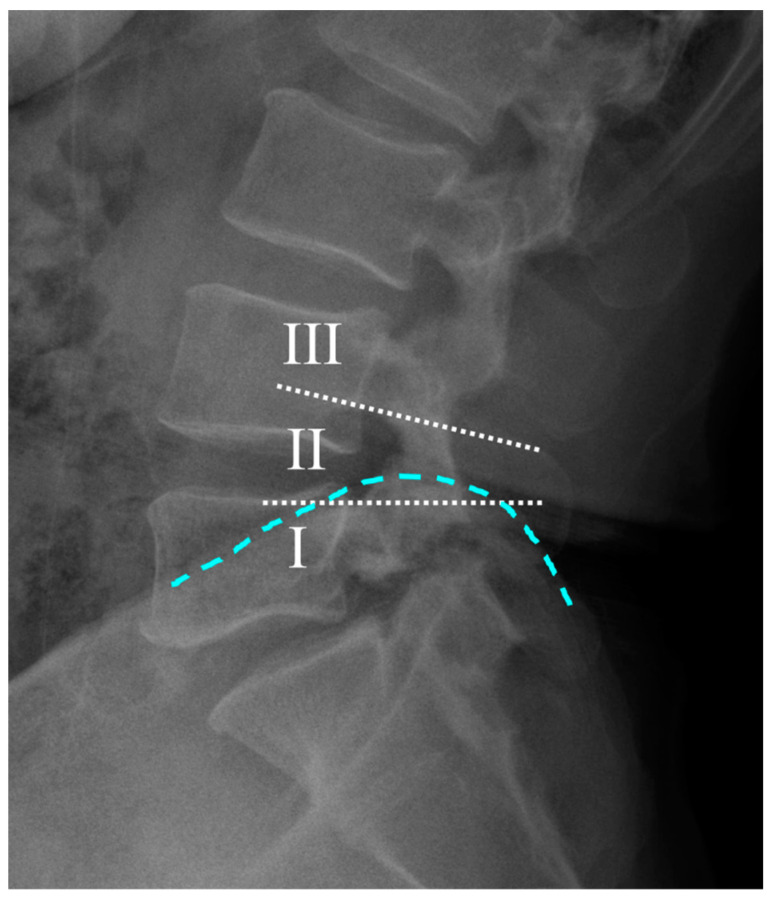
Method of categorizing crest levels into three groups based on the relationship between the height of the iliac crest (light blue dotted line) and the L4 and L5 pedicles on lumbar lateral radiographs. The white dotted lines represent the lower edge of the L4 pedicle and the upper edge of the L5 pedicle. I represents the iliac crest height below the upper edge of the L5 pedicle; II indicates the iliac crest height between the lower edge of the L4 pedicle and the upper edge of the L5 pedicle; and III represents the iliac crest height above the lower edge of the L4 pedicle.

This cohort study was approved by the institutional review board (IRB no. 2024AN0319; date of approval: 28 June 2024). Patient consent was waived due to the retrospective nature of the study. The study included patients who underwent single-level OLIF surgery at the L4–5 level for degenerative spinal stenosis between March 2019 and April 2022. All operations were performed by our senior author (J.H.Y.). Patients who received surgery for other conditions, such as infection, trauma, or tumors, and those with incomplete medical records were excluded. Only patients who had a minimum follow-up of 2 years, during which both patient questionnaires and radiological evaluations were completed, were included in the study. The remaining patients were categorized into three groups based on the height of the iliac crest relative to the L4 and L5 pedicles, as observed on plain lateral radiographs (Figure 1). The grading system for the iliac crest height was adapted from the method proposed by Song et al., which was validated in a previous study [9]. Groups I, II, and III represented different grades of iliac crest height. For each patient, demographic variables, including age, sex, height, weight, and comorbidities, were recorded.

### 2.2. Radiological Measurements

Radiological assessments included a comprehensive evaluation using plain radiography and computed tomography (CT). Lumbar lordosis and L4–5 segmental angles were measured on plain radiographs, and the presence of isthmic spondylolisthesis was assessed. The iliac crest was categorized based on its height on lumbar lateral plane radiographs (I, iliac crest below the L5 pedicle; II, iliac crest between the L4 and L5 pedicles; III, iliac crest above the L4 pedicle). Additionally, the relationship between the iliac crest height and the L4–5 disc level was quantified by measuring the distance from the midpoint of the L4–5 disc to the line connecting the ends of the iliac crest on lumbar AP radiographs.

**Figure 2:**
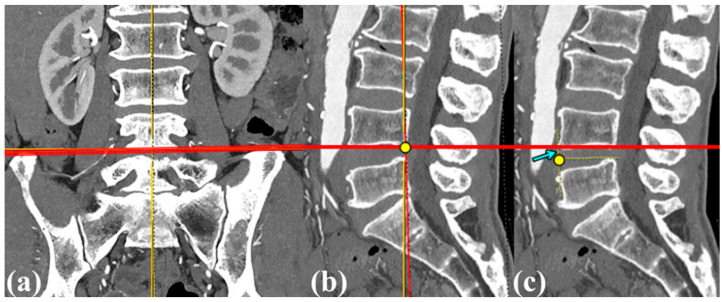
Measurement of the distance of the iliac crest in relation to the L4–5 disc space by computed tomography (CT). (**a**) The thick red line represents the connection between the highest points of both iliac crests in the CT coronal plane, with the midpoint of this line serving as a reference. (**b**) In the sagittal plane, the midpoint of the coronal plane’s red line is indicated by a yellow dot, which is then projected to create a horizontal red line. (**c**) The distance between the yellow dot marking the ventral side midpoint of the L4–5 disc space and the horizontal red line is measured as the distance between the iliac crest and the L4–5 disc space (light blue arrow).

The iliac crest height was also quantitatively assessed on coronal and sagittal CT images using the Picture Archiving and Communication System (PetaVision for Clinics, 3.1; Korea University Anam Hospital, Seoul, Republic of Korea). The assessment process first defined a tangential line touching both iliac crests in the coronal plane (Figure 2a). The red line serves as a reference for subsequent measurements. In Figure 2b,c, the minimum distance between this tangential line and the reference point in the L4–5 disc space was measured (sky blue arrow). To minimize potential rotational errors caused by the patient lying obliquely on the CT table, an axis passing through the ventral edge of the L4–5 disc space was established in the sagittal plane. This axis touches both iliac crests cranially and tangentially. The shortest distance from this line to the center of the ventral edge of the L4–5 disc space was evaluated as the iliac crest height relative to the L4–5 disc space. Additionally, CT scans were used to assess the presence of disc pathology, including the vacuum phenomenon and endplate sclerosis, and to measure both anterior and posterior disc heights.

### 2.3. Surgical Outcome Assessment

**Figure 3:**
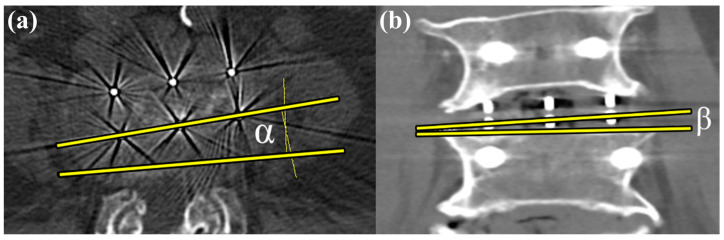
Measurement of cage insertion angles. (**a**) In the axial plane, the angle (α) formed between the long axis of the disc space and the long axis of the cage, measured using radiographic markers, is defined as the axial plane insertion angle. Positive angles are assigned to cages inserted from the left anterior to the right posterior position. (**b**) In the coronal plane, the angle (β) formed between the line parallel to the L5 upper endplate and the long axis of the cage, measured using radiographic markers, is defined as the coronal plane insertion angle. Negative angles are assigned to cages inserted from the left cranial to the right caudal direction.

This study evaluated various surgical outcomes following OLIF at the L4–5 disc space. The cage profile used during surgery, including its dimensions and angles, was documented. The occurrence of subsidence was assessed and graded according to the established criteria. The location of subsidence was categorized as L4 endplate, L5 endplate, or both. The study also recorded adverse outcomes, such as vessel injury during surgery. The success of fusion was evaluated using the Bridwell classification system. Postoperative CT was used to measure the cage insertion angles in both the coronal and axial planes. In the axial plane, positive angles were assigned to the cages inserted from the left anterior to the right posterior position (Figure 3a). In the coronal plane, negative angles were assigned to the cages inserted from the left cranial to the right caudal direction (Figure 3b). The postoperative anterior and posterior disc heights were also measured.

### 2.4. Clinical Outcomes

Clinical outcomes were assessed using several metrics. Back pain and leg pain were evaluated using a visual analog scale (VAS). Functional outcomes were measured using Oswestry Disability Index (ODI) and EuroQol-5 Dimension (Eq5D) scores. These outcomes were recorded preoperatively and at postoperative follow-up at 3 months, 6 months, 1 year, and 2 years.

### 2.5. Statistical Analyses

Statistical analyses were conducted to identify the predictors of subsidence following OLIF L4–5 procedures. Logistic regression analysis was performed to explore the relationship between the iliac crest height and the likelihood of subsidence. A receiver operating characteristic (ROC) curve analysis was also conducted to determine the cutoff value for iliac crest height that optimally predicts subsidence, and the area under the curve (AUC) and specific cutoff values were reported.

## 3. Results

### 3.1. Demographic and Radiographic Characteristics

Table 1 presents the demographic and radiographic characteristics of the patients across the three groups, categorized by iliac crest level. No significant differences in age, sex, height, weight, or the presence of comorbidities such as hypertension, diabetes, smoking, psychotic disease, liver disease, or pulmonary disease were observed among the three groups. However, significant differences in the iliac crest height, measured using plain radiography and CT, were noted among the three groups (*p* < 0.001). Additionally, the degree of lumbar lordosis significantly varied among the groups (*p* = 0.016).

### 3.2. Radiographic Results

**Figure 4:**
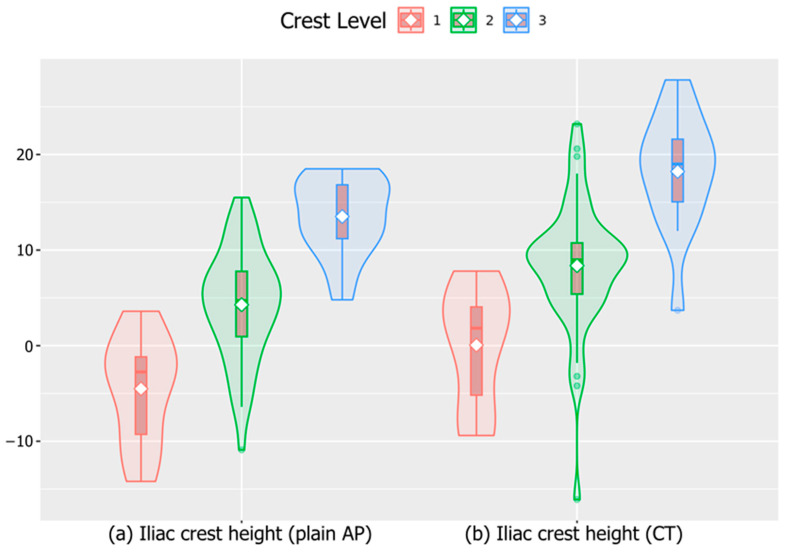
Quantitative comparison of iliac crest height among the three crest level groups. (**a**) Distance measured from the highest points of the iliac crest to the midpoint of the L4–5 disc using plain AP radiographs. (**b**) Distance measured between the iliac crest height and midpoint of the L4–5 disc space by CT. Both measurements showed significant differences across the three groups, with *p* < 0.001. AP, anteroposterior; CT, computed tomography.

**Figure 5:**
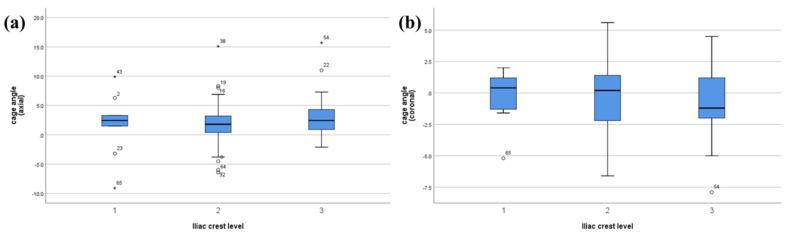
Comparison of cage insertion angles among the three crest level groups in the (**a**) axial and (**b**) coronal planes.

Analysis of the iliac crest height, categorized into three groups, demonstrated significant differences in measurements by both plane lumbar anteroposterior (AP) radiography and CT (Table 1 and Figure 4). Specifically, the iliac crest height increased progressively across the groups from crest level I to crest level III, with statistical significance noted on both plain radiography (*p* < 0.001) and CT (*p* < 0.001). However, the axial or coronal angles did not significantly differ among the three groups (Figure 5).

### 3.3. Surgical Outcomes

**Figure 6:**
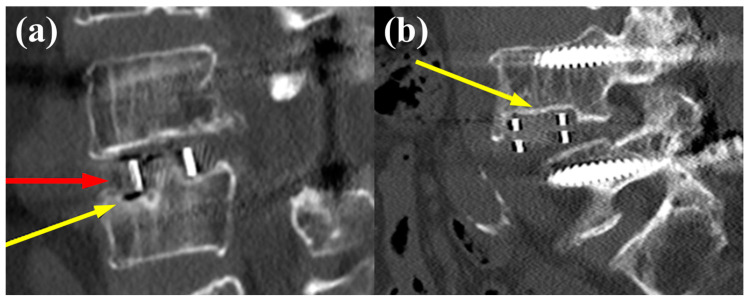
Typical cases of postoperative cage subsidence following OLIF at the L4–5 level. (**a**) Subsidence at the L5 upper endplate (yellow arrow) with a decrease in disc height and air accumulation at the graft site, suggesting non-union (red arrow). (**b**) Subsidence occurring at the L4 lower endplate (yellow arrow).

The analysis of surgical outcomes among the three groups indicated no significant differences in cage profile parameters, such as cage angle, cage height, postoperative anterior disc height, or postoperative posterior disc height (Table 2). However, subsidence was significantly more common in the crest level III group than in the other groups (*p* = 0.01). Moreover, the location of subsidence varied significantly among the groups (*p* = 0.016), with most of the subsidence occurring at the L5 endplate at crest level III. Typical cases of cage subsidence occurring at the L4 and L5 endplates are depicted in Figure 6. These representative images illustrate subsidence at the L5 upper endplate and the L4 lower endplate, highlighting the reduction in disc height, with non-union also observed in some cases (Figure 6a).

### 3.4. Patient-Reported Outcomes

Table 3 presents a comparison of the patient-reported outcomes across the three groups. Although no significant differences in the VAS scores for back and leg pain, ODI, or Eq5D were observed across most time points, a significant difference in VAS scores for back pain was observed at the 2-year follow-up (*p* = 0.021), with the crest level III group showing the highest pain scores among the groups.

### 3.5. Logistic Regression Analysis and ROC Curves

Table 4 summarizes the results of the logistic regression analysis performed to identify the predictors of subsidence in OLIF L4–5 procedures. Univariate analysis identified the cage axial angle (*p* = 0.03) and cage coronal angle (*p* = 0.049) as significant predictors of subsidence. Multivariate analysis further highlighted the iliac crest height measured by CT as a significant predictor of subsidence (*p* = 0.042).

**Figure 7:**
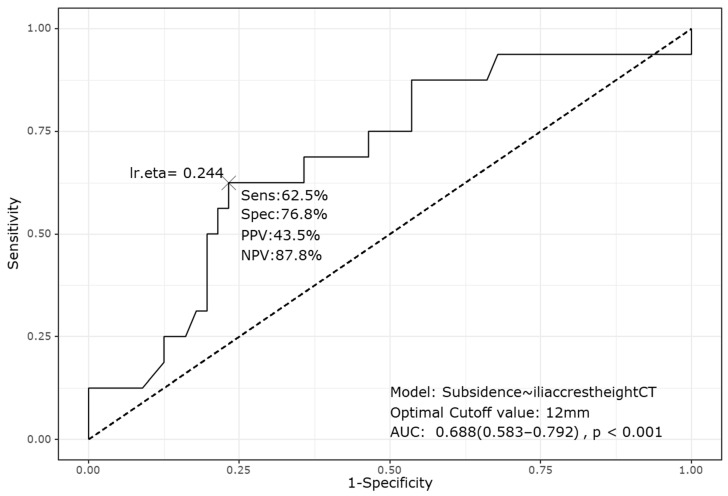
Receiver operating characteristic curve analysis of the relationship between the iliac crest height measured by CT and subsidence. The analysis identified a cutoff value of 12 mm, with an AUC of 0.688 (95% confidence interval: 0.583–0.792), sensitivity of 62.5%, and specificity of 76.8%. CT, computed tomography; AUC, area under the curve; PPV, positive predictive value; NPV, negative predictive value.

Additionally, the ROC analysis based on the logistic regression results identified the iliac crest height measured by CT as a significant factor associated with subsidence, yielding an AUC of 0.688. The analysis determined a cutoff value of 12 mm, indicating that an iliac crest height >12 mm measured by CT is associated with a higher likelihood of subsidence in L4–5 procedures (Figure 7).

## 4. Discussion

The core finding of our study was that when analyzing the outcomes of OLIF at the L4–5 level by categorizing patients based on the height of the iliac crest, the subsidence rate was significantly different across height grades. Specifically, although cage insertion obliquity showed no significant differences across the axial and coronal planes (Table 2), suggesting that such technical challenges can potentially be overcome by the surgeon’s skill, the subsidence rate markedly increased with a higher iliac crest height. This finding is particularly relevant because although surgeons may overcome the technical challenge of inserting the cage at an appropriate angle, the structural integrity of the vertebrae may remain compromised in patients with a high iliac crest, leading to a higher incidence of subsidence [1,4,7,8,10]. In crest level III, the subsidence rate was high at 43% (Table 2). Additionally, these subsidence cases were predominantly associated with damage to the L5 endplate, with a subsidence rate of 83% (10 of 12 cases) at crest level III. Thus, the anatomical positioning of the iliac crest may predispose the cage to impact the L5 endplate when inserted in an oblique direction from the anterior cranial to posterior caudal orientation [1,5,7].

Cage obliquity can cause several complications, such as radiculopathy due to the incorrect positioning of a large cage, spillage of the bone substitute placed in the cage, coronal malalignment, and pseudoarthrosis due to an increased gap between the cage and vertebral endplate [1,5,6,7,8]. These concerns highlight the importance of achieving the correct cage placement during OLIF. However, our study demonstrated that the iliac crest height did not directly influence cage obliquity, as evidenced by the lack of significant differences in axial and coronal cage insertion angles among the groups. Additionally, the absence of significant differences in the postoperative segmental angle, lordotic angle, and leg pain VAS further confirmed that obliquity did not vary based on the iliac crest height [5,11]. Given this finding, it would also be valuable to explore whether the relationship between iliac crest height and subsidence persists in other types of lumbar fusion surgeries, such as ALIF and PLIF, where cage obliquity is generally less of a concern. Investigating the influence of iliac crest height on subsidence in these alternative surgical approaches could deepen our understanding of the factors contributing to subsidence beyond the unique challenges presented by OLIF.

In this study, the occurrence of subsidence has significant clinical implications. The patients with subset of foraminal height can exacerbate nerve compression, leading to worsening leg pain as well as contributing to central stenosis [1,12,13]. The clinical significance of these findings underscores the importance of preventing subsidence to avoid these adverse outcomes. Previous studies have suggested that subsidence may be associated with endplate sclerosis, as Modic changes or endplate degeneration can affect the risk of postoperative subsidence [14,15]. However, in our study, no significant correlation between endplate sclerosis and subsidence was observed (*p* = 0.102), indicating that other factors may play a more prominent role in the development of subsidence in our cohort.

Another notable finding in our study was the correlation between iliac crest height and lumbar lordosis. A high iliac crest level was associated with increased lumbar lordosis (*p* = 0.016; Table 1). This raises an interesting question regarding the relationship between lumbar lordosis and iliac crest height and whether a higher iliac crest might contribute to greater lordosis, or whether this finding is purely incidental. Further research is needed to explore this association and to understand its implications. However, studies directly investigating this relationship are scarce, suggesting that our findings are among the first to report this association. Lumbar lordosis generally follows the pelvic incidence, and patients with a high pelvic incidence often have an L5–S1 level situated deep within the pelvis [8,16]. This could potentially relate to the distance between the L4–5 level and the iliac crest height, indicating a possible connection between these anatomical factors.

Our study highlights the reliability of combining categorical and quantitative analyses to evaluate the relationship between iliac crest height and OLIF outcomes [17,18,19]. By categorizing patients based on the iliac crest height and by utilizing quantitative measurements from plain lumbar AP radiographs and CT scans, we elucidated how iliac crest height affects surgical outcomes. Our quantitative analysis, particularly the univariate and multivariate logistic regression analyses, identified the iliac crest height measured by CT as the most significant predictor of subsidence (*p* = 0.042; Table 4). Based on these results, the ROC analysis determined a cutoff value of 12 mm, indicating that an iliac crest height > 12 mm significantly increased the likelihood of subsidence following OLIF at the L4–5 level (Figure 6). This finding aligns with the qualitative observation that crest level III, where the mean iliac crest height was approximately 18 mm, had a notably higher subsidence rate of 43% than the other crest levels. The consistent results from both the grading and quantitative analyses enhance the credibility of our study, as they converge on a reliable and robust conclusion. Given these findings, it is likely that a higher iliac crest height could increase the risk of intraoperative endplate damage, even though no significant differences were observed in cage insertion angles across the groups.

Considering these findings, patients with an iliac crest height ≥ 12 mm, which is classified as crest level III, are at a higher risk for subsidence than patients with a lower iliac crest height. The negative outcomes associated with subsidence, including potential nonunion and the need for revision surgery, emphasize the importance of meticulous preoperative planning in these patients [20]. Surgeons should carefully consider the anatomical challenges posed by a high iliac crest. Moreover, employing alternative strategies such as using a cage that allows for insertion at a more diagonal angle, similar to that used in L5–S1 OLIF, or modifying the surgical approach to minimize the risk of subsidence may be necessary [4,10,11,21].

### Limitations

First, this study had a retrospective design, which may have introduced a selection bias and limited the generalizability of our findings. Second, the sample size, particularly in the high iliac crest height group, was relatively small, which may have affected the statistical power of the analyses. Third, although we identified a significant association between iliac crest height and subsidence, other factors such as bone quality or the specific surgical technique used, which may also play a role, were not fully considered in our analysis. Finally, our follow-up period, which was adequate for assessing short- to mid-term outcomes, may not have fully captured the long-term effects of subsidence on spinal fusion success and overall patient outcomes. Future studies with larger sample sizes, prospective designs, and longer follow-up periods are needed to validate our findings and to further explore the complex interplay between the iliac crest height and OLIF outcomes.

## 5. Conclusions

The iliac crest height plays a significant role in the outcomes of OLIF at the L4–5 level. Although the iliac crest height does not affect cage obliquity, it is strongly associated with an increased risk of subsidence, particularly in patients with an iliac crest height ≥ 12 mm. These findings underscore the importance of meticulous preoperative planning and the need for alternative surgical strategies to mitigate the risk of subsidence in patients with high iliac crests. Although our study provides valuable insights, further research with larger prospective cohorts is necessary to fully understand the long-term implications of iliac crest height on OLIF outcomes.

## Figures and Tables

**Table 1:** Demographic and radiographic characteristics of the three groups.

Parameter	Crest Level I (*n* = 20)	Crest Level II (*n* = 91)	Crest Level III (*n* = 28)	*p* Value
Demographic parameters				
Age (y)	71.6 ± 7.4	74.0 ± 7.4	74.8 ± 6.1	0.078
Sex, M:F	12:8	24:67	10:18	0.121
Height (cm)	157.2 ± 5.5	155.9 ± 7.9	157.8 ± 7.5	0.137
Weight (kg)	62.8 ± 10.3	61.4 ± 9.5	63.3 ± 11.01	0.208
BMD	−1.2 ± 1.8	−1.5 ± 1.4	−1.4 ± 2.1	0.752
HTN, *n*	7	29	11	0.194
DM	4	17	7	0.095
Smoking	5	22	15	0.137
Psychotic disease	2	1	6	0.266
Liver disease	0	3	8	0.863
Pulmonary disease	1	5	7	0.342
Radiographic parameters				
Iliac crest height, mm (plain radiograph)	−1.8 ± 2.8	4.3 ± 5.9	13.3 ± 4.3	<0.001 *
Iliac crest height, mm (CT)	0.1 ± 6.2	8.4 ± 6.8	18.2 ± 6.1	<0.001 *
Lumbar lordosis, °	23.6 ± 13.1	34.2 ± 12.3	37.6 ± 14.4	0.016 *
L4−5 segmental angle, °	12.0 ± 6.2	14.0 ± 7.3	16.2 ± 8.2	0.348
Isthmic spondylolisthesis	2 (8.3%)	6 (6.5%)	0 (0.0%)	0.339
Vacuum phenomenon	14 (58.3%)	70 (76.1%)	20 (71.4%)	0.223
Endplate sclerosis	11 (55.0%)	66 (72.5%)	17 (60.7%)	0.216
Preop ADH, mm	7.1 ± 1.1	7.1 ± 2.9	8.1 ± 2.9	0.462
Preop PDH, mm	4.2 ± 0.9	4.2 ± 2.1	3.9 ± 1.9	0.925

HTN, hypertension; DM, diabetes mellitus; CT, computed tomography; ADH, anterior disc height; PDH, posterior disc height; BMD, bone mineral density. * *p* < 0.05.

**Table 2:** Surgical results of the three groups.

	Crest Level I (*n* = 20)	Crest Level II (*n* = 91)	Crest Level III (*n* = 28)	*p* Value
Cage profile				
Cage angle	6.7 ± 2.4	6.8 ± 2.1	7.8 ± 2.8	0.111
Cage height	10.7 ± 1.0	11.0 ± 1.2	11.8 ± 1.7	0.084
Cage angle (axial)	1.9 ± 5.1	1.9 ± 3.8	3.6 ± 4.8	0.39
Cage angle (coronal)	−0.2 ± 2.1	−0.4 ± 2.6	−0.9 ± 3.1	0.793
Postop ADH	10.9 ± 2.1	10.6 ± 1.9	11.1 ± 1.6	0.698
Postop PDH	6.4 ± 1.7	7.4 ± 1.7	6.3 ± 1.5	0.092
Lumbar lordosis, °	26.6 ± 11.3	35.7 ± 14.1	36.5 ± 12.4	0.163
L4–5 segmental angle, °	13.6 ± 5.8	15.2 ± 5.3	17.0 ± 11.7	0.472
Subsidence	2 (10%)	18 (19%)	12 (43%)	0.01 *
Subsidence location				0.016 *
L5 endplate	2	12	8	
L4 endplate		6	2	
Both endplates			2	
Complete fusion	12 (60.0%)	48 (57.1%)	12 (46.2%)	0.556
Bridwell fusion grade	2.3 ± 0.9	2.2 ± 0.7	2.3 ± 0.6	0.845
Vessel injury		1	1	0.503
Revision			2	0.124

ADH, anterior disc height; PDH, posterior disc height. * *p* < 0.05.

**Table 3:** Comparison of patient-reported outcomes among the three groups.

		Crest Level I (*n* = 20)	Crest Level II (*n* = 91)	Crest Level III (*n* = 28)	*p* Value
Back pain VAS	Preoperative	7.4 ± 1.3	6.3 ± 2.1	7.4 ± 1.0	0.085
	Postop 3M	3.1 ± 2.5	3.1 ± 1.9	3.1 ± 1.7	0.990
	Postop 6M	2.6 ± 2.0	2.3 ± 1.6	2.6 ± 1.5	0.481
	Postop 1Y	2.8 ± 1.6	2.3 ± 1.5	2.9 ± 1.6	0.503
	Postop 2Y	3.0 ± 3.2	2.4 ± 1.4	4.6 ± 2.8	0.021 *
Leg pain VAS	Preoperative	5.9 ± 1.4	5.4 ± 2.1	5.3 ± 2.1	0.531
	Postop 3M	1.9 ± 1.5	2.2 ± 1.7	1.9 ± 1.2	0.476
	Postop 6M	2.4 ± 2.0	1.7 ± 1.4	1.6 ± 1.1	0.072
	Postop 1Y	2.0 ± 1.7	1.6 ± 1.3	1.5 ± 1.0	0.404
	Postop 2Y	2.6 ± 1.6	1.9 ± 1.6	1.8 ± 2.3	0.449
ODI	Preoperative	64.0 ± 6.5	63.9 ± 14.2	64.9 ± 15.2	0.972
	Postop 3M	49.1 ± 10.9	49.3 ± 11.6	44.1 ± 17.2	0.436
	Postop 6M	43.4 ± 16.8	35.7 ± 13.3	34.9 ± 15.3	0.236
	Postop 1Y	35.7 ± 17.0	31.6 ± 13.7	32.3 ± 15.6	0.731
	Postop 2Y	36.5 ± 19.0	30.6 ± 13.5	39.5 ± 23.1	0.438
Eq5D	Preoperative	15.2 ± 1.7	15.9 ± 3.2	16.0 ± 2.1	0.828
	Postop 3M	11.8 ± 1.6	12.0 ± 3.0	11.1 ± 2.9	0.697
	Postop 6M	10.0 ± 2.4	9.9 ± 2.4	10.0 ± 2.4	0.986
	Postop 1Y	9.5 ± 3.1	9.3 ± 2.5	9.6 ± 2.5	0.932
	Postop 2Y	10.7 ± 4.7	9.2 ± 2.8	11.8 ± 4.5	0.253

VAS, visual analog scale; ODI, Oswestry Disability Index; Eq5D, EuroQol-5 Dimension. * *p* < 0.05.

**Table 4:** Logistic regression analysis for predicting subsidence in OLIF L4–5 procedures.

Univariate Analysis							
	Beta	SE	z Value	*p* Value	OR	lcl	ucl
Lumbar lordosis	0.0285	0.0223	1.28	0.202	1.03	0.99	1.08
L4–5 segmental angle	−0.0051	0.0392	−0.13	0.896	0.99	0.92	1.07
Cage angle	−2.9131	319.4502	−0.01	0.993	0.05		
Cage height	−0.1179	0.2281	−0.52	0.605	0.89	0.55	1.37
Iliac crest height (AP)	0.0641	0.0407	1.58	0.115	1.07	0.99	1.16
Iliac crest height (CT)	0.0679	0.0375	1.81	0.07	1.07	1	1.16
ADH	−0.1303	0.1144	−1.14	0.255	0.88	0.69	1.09
PDH	−0.1565	0.1648	−0.95	0.342	0.86	0.6	1.16
Cage axial angle	0.1665	0.0769	2.17	0.03	1.18	1.03	1.4
Cage coronal angle	−0.23	0.1168	−1.97	0.049	0.79	0.62	0.99
Endplate sclerosis	0.9461	0.5844	1.62	0.105	2.58	0.84	8.54
Multivariate analysis							
Cage axial angle	0.127	0.0633	2.01	0.055	1.14	1.01	1.3
Iliac crest height (CT)	0.091	0.0584	0.16	0.042 *	1.093	1	1.19
Residual deviance/df = 115.2/126 = 0.91, pseudo-R^2^ = 0.36 (Nagelkerke)							

SE, standard error; OR, odds ratio; lcl, lower confidence interval; ucl, upper confidence interval; AP, plane lumbar anteroposterior radiograph; CT, computed tomography; ADH, anterior disc height; PDH, posterior disc height. * *p* < 0.05.

## Data Availability

The data presented in this study are available on request from the corresponding author.
